# Genome-scale transcriptomic insights into the gene co-expression network of seed abortion in triploid *Siraitia grosvenorii*

**DOI:** 10.1186/s12870-022-03562-4

**Published:** 2022-04-05

**Authors:** Rongchang Wei, Dongping Tu, Xiyang Huang, Zuliang Luo, Xiaohua Huang, Nan Cui, Juan Xu, Faqian Xiong, Haifeng Yan, Xiaojun Ma

**Affiliations:** 1 Cash Crops Research Institute, Guangxi Academy of Agricultural Sciences, Nanning, 530007 China; 2 Guangxi University of Chinese Medicine, Nanning, 530020 China; 3 Guangxi Institute of Botany, Guangxi Zhuang Autonomous Region and Chinese Academy of Sciences, Guangxi Key Laboratory of Plant Functional Phytochemicals Research and Sustainable Utilization, Guilin, 541006 China; 4 Institute of Medicinal Plant Development, Chinese Academy of Medical Sciences & Peking Union Medical College, Beijing, 100193 China; 5 Biotechnology Research Institute, Guangxi Academy of Agricultural Sciences, Nanning, 530007 China; 6 Sugarcane Research Institute, Guangxi Academy of Agricultural Sciences, Nanning, 530007 China

**Keywords:** *Siraitia grosvenorii*, Seed abortion, Transcriptome, Gene-regulatory network, Plant hormones

## Abstract

**Background:**

*Siraitia grosvenorii* (Swingle) C. Jeffrey, also known as Luohanguo or monk fruit, is a famous traditional Chinese medicine ingredient with important medicinal value and broad development prospects. Diploid *S. grosvenorii* has too many seeds, which will increase the utilization cost of active ingredients. Thus, studying the molecular mechanism of seed abortion in triploid *S. grosvenorii*, identifying the abortion-related genes, and regulating their expression will be a new direction to obtain seedless *S. grosvenorii*. Herein, we examined the submicroscopic structure of triploid *S. grosvenorii* seeds during abortion.

**Results:**

Upon measuring the endogenous hormone content, we found that abscisic acid (ABA) and trans-zeatin (ZR) levels were significantly downregulated after days 15 and 20 of flowering. RNA sequencing of triploid seeds at different developmental stages was performed to identify key genes regulating abortion in triploid *S. grosvenorii* seeds. Multiple genes with differential expression between adjacent stages were identified; seven genes were differentially expressed across all stages. Weight gene co-expression network analysis revealed that the enhancement of monoterpene and terpene metabolic processes might lead to seed abortion by reducing the substrate flow to ABA and ZR.

**Conclusions:**

These findings provide insights into the gene-regulatory network of seed abortion in triploid *S. grosvenorii* from different perspectives, thereby facilitating the innovation of the breeding technology of *S. grosvenorii*.

**Supplementary Information:**

The online version contains supplementary material available at 10.1186/s12870-022-03562-4.

## Background

*Siraitia grosvenorii* (Swingle) C. Jeffrey is a species of the genus *Siraitia* Merr. (Cucurbitaceae). It is native to the southern parts of China and is mainly found in Guangxi Province [1]. *S. grosvenorii* is also known as Luohanguo (LHG) or monk fruit. LHG is not only used as a food ingredient, but also as in traditional Chinese medicine. In China, LHG has been used as a natural cough suppressant and expectorant for over 300 years and is one of the first approved medicinal food homology species. It is also highly favored internationally [1, 2].

Because LHG contains sweet glycosides that are naturally low in calories, the extracts from ripe LHG can be used as supplements and sweeteners in sugar-free health foods and beverages [3, 4]. In addition, this plant is currently used as an analgesic in the treatment of lung injury and an emollient for the treatment of sore throat, thirst, and constipation [5]. The crude extracts and purified products of *S. grosvenorii* have several biological functions, including immunological, antioxidant, anti-tussive, sputum-reducing, hypoglycemic, hepatoprotective, and antimicrobial activities [5–9]. Previous studies have revealed that several different classes of compounds were isolated from *S. grosvenorii*, including triterpenoids, iridoid, flavonoids glycosides, vitamins, proteins, saccharides, and a volatile oil [10–12]. The triterpene compounds, mogrosides, are considered the main active ingredients of *S. grosvenorii*. And mogroside V is the main component contributing to the sweet taste [13, 14]. With the development of science and technology, the application of *S. grosvenorii* in the field of medicine can be further explored. Therefore, *S. grosvenorii* is a natural product with high development potential for medicine and commodity production, and it is attracting increasing attention from scientific research community and commercial establishments [10, 15].

However, at present, there is no effective method to synthesize mogroside V, which completely depends on artificial cultivation to obtain raw materials. And it is mainly distributed in pulp, and almost absent in seeds [16]. The seeds of diploid *S. grosvenorii* fruit account for 40% ~ 50% of the fresh weight of the fruit. The extremely low content of mogroside V and large number of lipid components in the seeds increase the utilization cost and the purification difficulty of mogroside V. Seed removal is an effective way to improve extraction efficiency and reduce costs. However, it is difficult to implement artificial seed removal in production due to high labor costs. Therefore, the cultivation of seedless *S. grosvenorii* fruit with high content of mogroside V has become a key topic in the breeding of *S. grosvenorii* [17].

In the past ten years, chromosome engineering has been a hot topic in *S. grosvenorii* germplasm research, and several polyploid *S. grosvenorii* resources have been obtained. The tetraploid female plants obtained via colchicine induction were hybridized with the diploid male parent to obtain triploid seeds, which produced an excellent triploid *S. grosvenorii* germplasm resource. Compared with the diploid *S. grosvenorii*, the triploid plants not only had larger vegetative and reproductive organs but also seedless or almost seedless fruits. Furthermore, the mogroside V content in triploid fruits was 36.28% higher than that in control fruits, and the triploid plants exhibited stronger stress resistance [18, 19].

Seed development can be divided into three major phases: embryogenesis, seed maturation, and desiccation. Embryogenic processes include cell division and expansion, morphogenesis, and the beginning of the endosperm and embryo development. A series of morphological, physiological, and biochemical changes are involved in seed development [20–22]. The production of viable seeds is thought to require the co-regulation of multiple plant endogenous hormones. Abscisic acid (ABA) and gibberellin (GA) are the main hormones that regulate seed formation. The process of seed dormancy interruption is determined by the distribution of GA and ABA. In addition, ABA regulates seed maturation, embryo morphogenesis, and desiccation [23]. Auxin is an important molecule that regulates seed development in conjunction with ABA [24–27]. Trans-zeatin (ZR) is a cytokinin that exists in seeds that can induce cell division and proliferation [28]. We speculate that seed abortion may be regulated by fluctuations in the abovementioned four hormones; however, related evidence is absent.

At present, there are few reports on breeding new species of *S. grosvenorii* using genetic-engineering technology. Li et al. cloned a pathogenesis-related gene from *Arabidopsis thaliana* and introduced it into *Momorrhoea grossida* via the *Agrobacterium*-mediated method to obtain transgenic *S. grosvenorii* with resistance to tobacco mosaic virus [29]. Additionally, using the *Agrobacterium*-mediated method and an overexpression vector constructed using the fruit-specific promoter 2*A*11 and gene *iaaM*, which encodes tryptophan monooxygenase, a key enzyme for auxin synthesis, Zhou et al. obtained five positive plants that blossomed normally in the field and showed parophytic fruiting [30]. Thus, studying the molecular mechanisms of seed abortion and identifying the genes and pathways involved may provide a theoretical basis for genetically engineering a new seedless *S. grosvenorii* resource. Here, we analyzed the transcriptome sequencing data of triploid seeds of *S. grosvenorii* at different developmental stages and then combined this information with the endogenous hormonal changes during seed development to reveal the gene-regulatory network involved in the formation of triploid *S. grosvenorii* abortive seeds. Genes involved in multiple pathways related to ABA and ZR, which contribute to seed abortion, were identified. These genes could be candidates for breeding seedless *S. grosvenorii* fruits via genetic engineering.

## Results

### The profile of seed abortion in *S. grosvenorii*

**Fig. 1:**
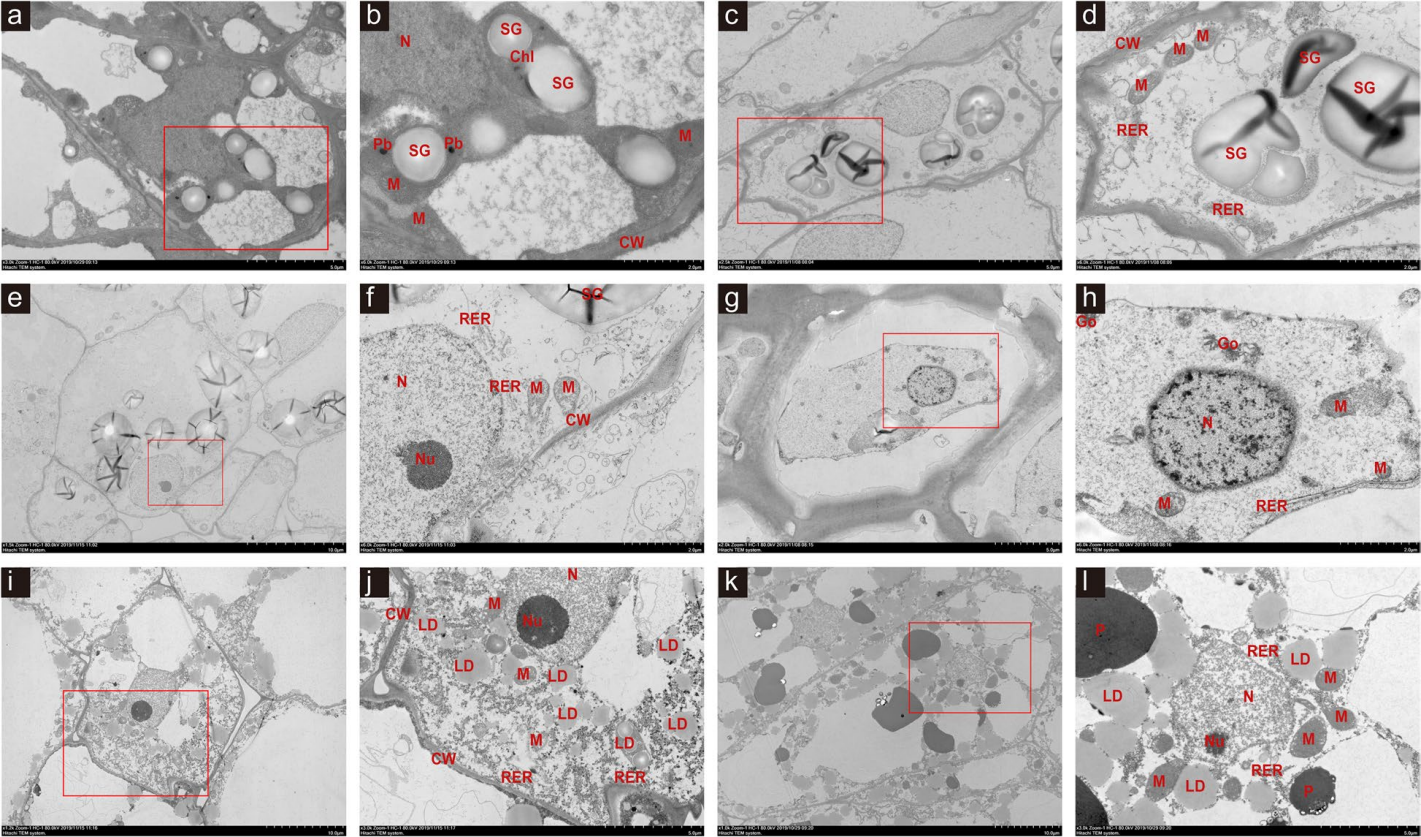
Transmission electron microscopy (TEM) images of the tissues with structural changes during seed abortion. The original (**a**, **c**, **e**, **g**, **i**, and **k)** and zoomed-in (**b**, **d**, **f**, **h**,**j**, and **l**) TEM images are shown for different stages, namely 5DAF (**a-b**), 10DAF (**c-d**), 15DAF (**e-f**), 20DAF (**g-h**), 25DAF (**i-j**), and 30DAF (**k-l**). Red frames in **a**, **c**, **e**, **g**, **i**, and **k** indicate zoomed-in regions in these images. N, Nu, M, V, Chl, CW, RER, SG, LD, Go, P, and Pb indicate nucleus, micronucleus, mitochondrion, vacuole, chloroplast, cell wall, rough endoplasmic reticulum, starch granule, lipid droplet, Golgi complex, proteosomes, and protein body respectively

The structural changes in the tissues at each stage were observed using transmission electron microscopy (TEM). There was no obvious edema in the cytoplasm and no obvious separation of the plasma wall at stage 5 days after flowering (DAF) (Fig. 1a and b). The nucleus was irregular, the mitochondria were oval, the crest and rough endoplasmic reticulum were moderately dilated, the surface ribosome was locally exfoliated, and the number of intracellular starch granules was abundant. Severe edema of the cytoplasm and slight separation of the plasma wall were observed at stages 10DAF (Fig. 1c and d) and 15DAF (Fig. 1e and f), respectively. Severe separation of the plasma wall, local damage to the cell membrane, and thickness of the cell wall were observed at 20DAF (Fig. 1g and h) and gradually recovery was observed from stages 25DAF (Fig. 1i and j) to 30DAF (Fig. 1k and l).

**Fig. 2:**
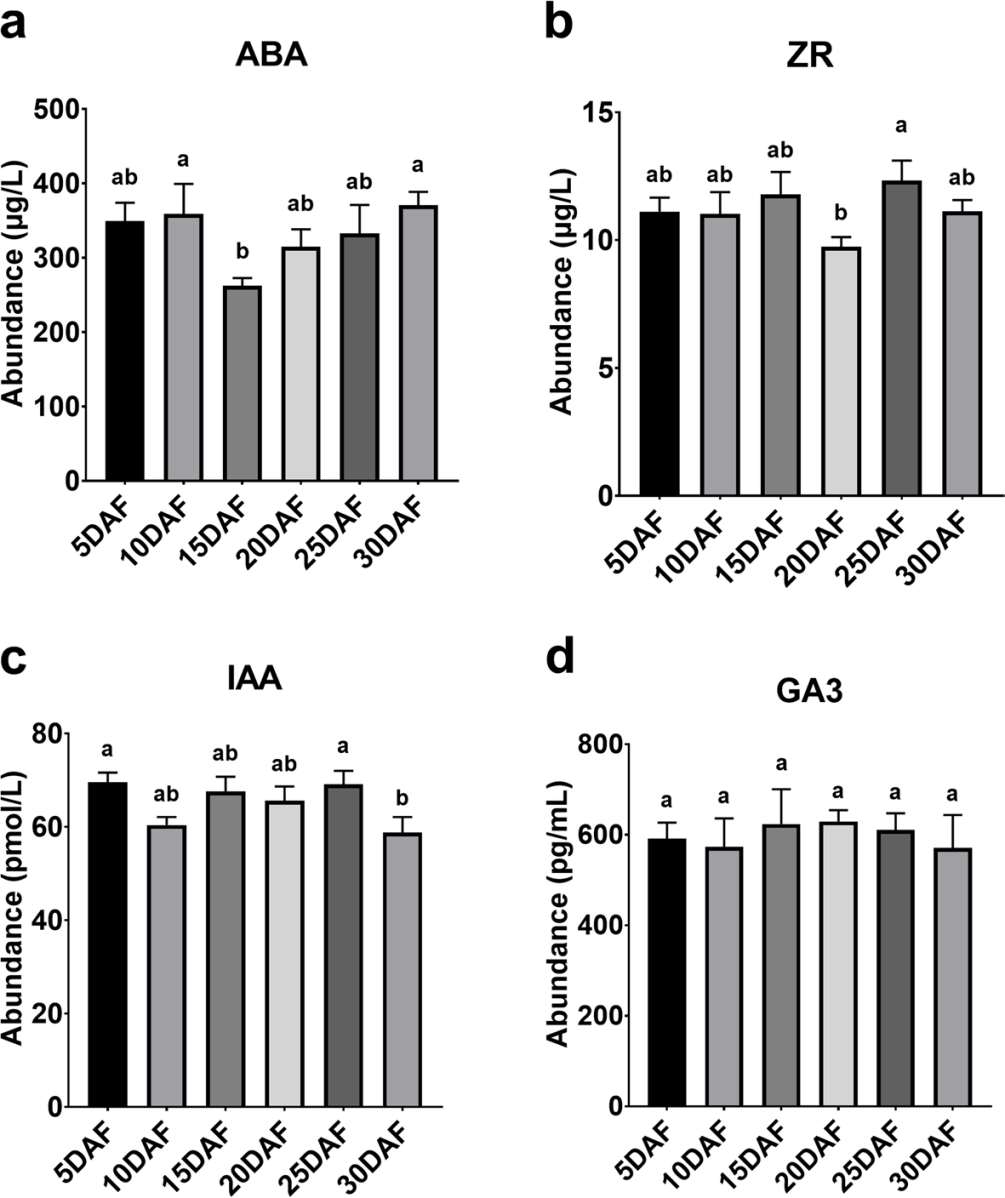
The levels of endogenous plant hormone during seed abortion: ABA (**a**), ZR (**b**), IAA (**c**), and GA3 (**d**). ABA, abscisic acid; ZR, trans-zeatin-riboside; IAA, indole-3-acetic acid; GA3, gibberellin A3. Data are presented as error bar plots showing the mean ± SE of three/four replicates. Different letters indicate significant differences at *p*-value < 0.05 (Least significance difference test)

We also examined the levels of endogenous plant hormones, ABA, ZR, indole-3-acetic acid (IAA), and gibberellin A3 (GA3), during seed abortion using enzyme-linked immunosorbent assay (ELISA) (Fig. 2a-d). Interestingly, the levels of ABA and ZR decreased remarkably at stages 15DAF and 20DAF, respectively. The above changes in submicroscopic structure and levels of endogenous plant hormones indicated that 15–20DAF was a key transition period during seed abortion. We will mainly focus on the exploration of the transcriptomic changes at stages 15DAF and 20DAF in the subsequent analyses.

### Global analysis of the RNA sequencing (RNA-seq) data

**Fig. 3:**
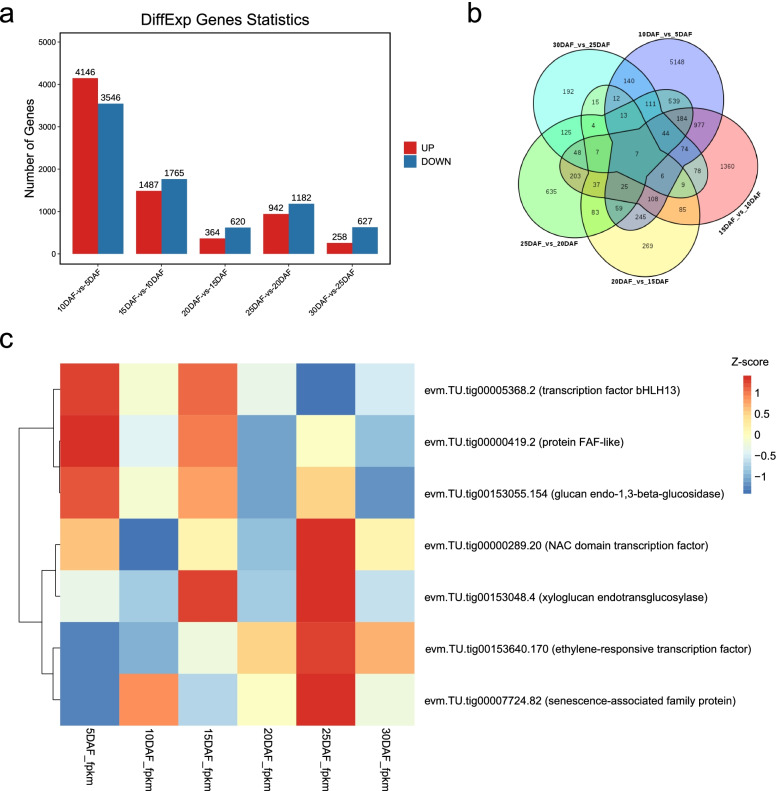
Differentially expressed genes (DEGs) in five comparisons between consecutive stages. **a** Barplot representing the number of upregulated (red) and downregulated (blue) genes between consecutive stages. The genes with |log2FC| > 1 and FDR-adjusted *p*-value < 0.05 were identified as significant DEGs. **b** Venn diagram of differentially expressed genes. **c** Heatmap showing 7 differentially expressed genes among all comparisons in **b**. The colorbar represents Z-score normalized fragments per kilobase of transcripts per million fragments mapped (FPKM)

The gene abundance was quantified by RNA-seq transcriptome analysis (Table S1). Principal component analysis (PCA) showed that the three replicates of each stage trended to cluster together (Fig. S1). Differentially expressed genes (DEGs) (|log2fold-change| > 1 and false discovery rate (FDR)-adjusted *p*-value < 0.05) were identified between consecutive stages (Tables S2, S3, S4, S5 and S6). The number of differentially expressed genes (DEGs) in the five comparisons is shown in Fig. 3a and b. The total DEGs in 10DAF vs 5DAF were 7692 (4145 upregulated and 3546 downregulated), 15DAF vs 10DAF were 3252 (1487 upregulated and 1765 downregulated), 20DAF vs 15DAF were 984 (364 upregulated and 620 downregulated), 25DAF vs 20DAF were 2124 (942 upregulated and 1182 downregulated), and 30DAF vs 25DAF were 885 (258 upregulated and 627 downregulated).

The DEGs were further functionally classified into gene ontology (GO) terms (Tables S7, S8, S9, S10 and S11; *p* < 0.05). DEGs in 20DAF vs. 15DAF were identified to be involved in meristem structural organization, morphogenesis of a branching structure, adaxial/abaxial axis specification, positive regulation of catalytic activity, oxidoreductase activity, and glucosidase activity (Table S9), whereas those in 25DAF vs. 20DAF were involved in trehalose metabolic process, seed germination, response to sucrose, transmembrane receptor histidine kinase activity, and so forth. (Table S10).

Seven common DEGs shared by all comparisons of adjacent stages were identified (Fig. 3b), which revealed fluctuations in expression levels across all stages (Fig. 3c). These DEGs were found to be associated with toxin metabolic processes, regulation of gene expression, glucan metabolic process, and external encapsulating structure organization. Overall, the dynamic landscape of triploid *S. grosvenorii* seed formation showed a highest number of DEGs during the early stages of seed formation and only a few shared DEGs across stages were observed.

### Identification of temporal expression trends across *S. grosvenorii* seed transcriptomes

**Fig. 4:**
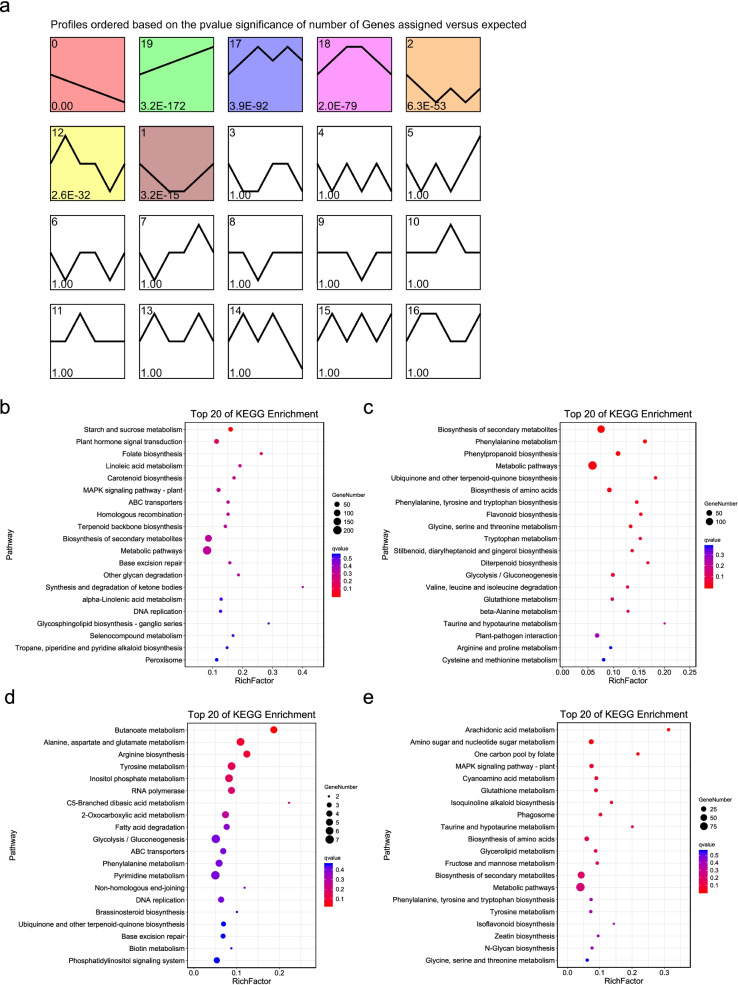
Temporal expression trends during seed abortion. **a** STEM analysis of the gene expression profiles. Each box indicates a model profile, and the colored profiles shown are significant. The numbers in the box provide the order of the profile (upper left) and the *p*-value indicates significance (lower left). **b-e** Top 20 pathways from KEGG enrichment analysis for the genes in profiles 18 (**b**), 0 (**c**), 19 (**d**), and 1 (**e**) in (**a**)

We performed a short time-series expression miner (STEM) analysis for the identified DEGs to visualize the expression patterns of these genes. As a result, seven expression profiles were found to be statistically significant (Fig. 4a). Profiles 0 and 19 tended to be continuously downregulated and upregulated during seed abortion, respectively, whereas profiles 1 and 18 reached an expression peak and valley at stages 15DAF and 20DAF, respectively.

Kyoto Encyclopedia of Genes and Genomes (KEGG) pathway enrichment analysis was performed for the four expression trends (19, 0, 1, and 18). The genes in profile 0 with a continuously downregulated trend were mainly enriched in starch and sucrose metabolism, plant hormone signal transduction, linoleic acid metabolism, replication and repair, and other glycan degradation (Fig. 4b), whereas those in profile 19, which were continuously upregulated, were related to amino acid metabolism; phenylpropanoid biosynthesis; ubiquinone and other terpenoid-quinone biosynthesis; flavonoid biosynthesis; stilbenoid, diarylheptanoid, and gingerol biosynthesis; diterpenoid biosynthesis; glycolysis/gluconeogenesis; and plant-pathogen interactions (Fig. 4c). The genes in profile 1 with the lowest expression level at 15DAF and 20DAF were involved in RNA polymerase, butanoate metabolism, inositol phosphate metabolism, and fatty acid degradation (Fig. 4d). Finally, the genes in profile 18 with the highest expression levels at stages 15DAF and 20DAF were involved in arachidonic acid metabolism, amino sugar and nucleotide sugar metabolism, one carbon pool by folate, cyanoamino acid metabolism, glutathione metabolism, isoquinoline alkaloid biosynthesis, phagosome, glycerolipid metabolism, and fructose and mannose metabolism (Fig. 4e). These results suggest that these pathways may play important roles during seed abortion in *S. grosvenorii*.

### Co-expression network associated with seed abortion

**Fig. 5:**
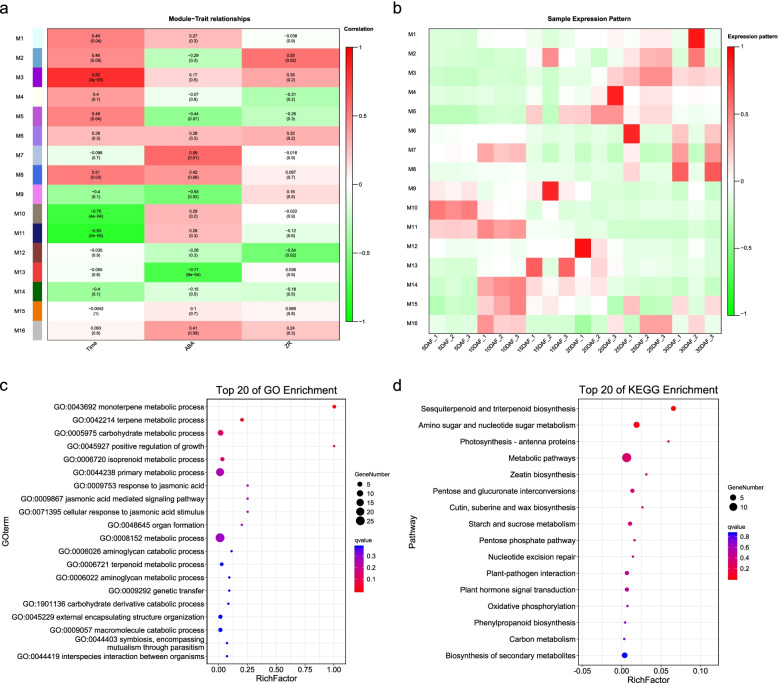
WGCNA and characteristics of the modules with different colors. **a** The association between the module eigengene E and the traits of development stages and levels of ABA and ZR, respectively. The correlation coefficient is presented in each row, and the FDR-adjusted *P*-value is shown in parentheses. The color bar represents correlation coefficient value. **b** Heatmap depicting the expression pattern of the WGCNA modules for each sample. Red indicates high expression, and green indicates low expression. **c** Top 20 pathways from GO enrichment analysis for the genes in the M13 module. **d** Top 20 pathways from KEGG enrichment analysis for the genes in the M13 module

Some genes that did not show significantly differential expression under the threshold of differentially expressed analysis might also play important regulatory roles in the key pathways [31]. To explore the global gene regulatory network, weighted gene co-expression network analysis (WGCNA) was used to cluster 21,152 genes after filtering those with low expression levels, resulting in 16 gene modules with different colors (Fig. S2 and Table S12). Combined with the module-trait relationship and module significance, we eventually identified the M13 module as the most phenotypically relevant module, which showed a negative correlation with the level of ABA (the correlation coefficient was − 0.71 and the FDR-adjusted *p*-value was 9e-04), whereas the M7 module showed a positive correlation (Fig. 5a). The M12 and M2 modules were also found to be negatively and positively associated with the level of ZR, respectively, with statistical significance (Fig. 5a).

We explored the expression profiles of each module across the different stages (Fig. 5b). Interestingly, we found that the genes in the M13 module were specifically, highly expressed at 15DAF and 20DAF (Fig. 5b), and most of the DEGs (58.82%) were belonged to profile 18 in temporal analysis. By combining the above results, we considered the M13 module to be negatively associated with the changes in ABA levels at 15DAF. According to the results of GO enrichment analysis, the genes in the M13 module were enriched in monoterpene metabolic processes and terpene metabolic processes (Fig. 5c). Additionally, KEGG pathway analysis revealed that the genes were involved in sesquiterpenoid and triterpenoid biosynthesis and amino sugar and nucleotide sugar metabolism (Fig. 5,5d). These findings indicate that these genes, which are related to terpene metabolism, are highly expressed at stage 15DAF and may play an important role in the decrease of ABA levels, thereby leading to seed abortion.

**Fig. 6:**
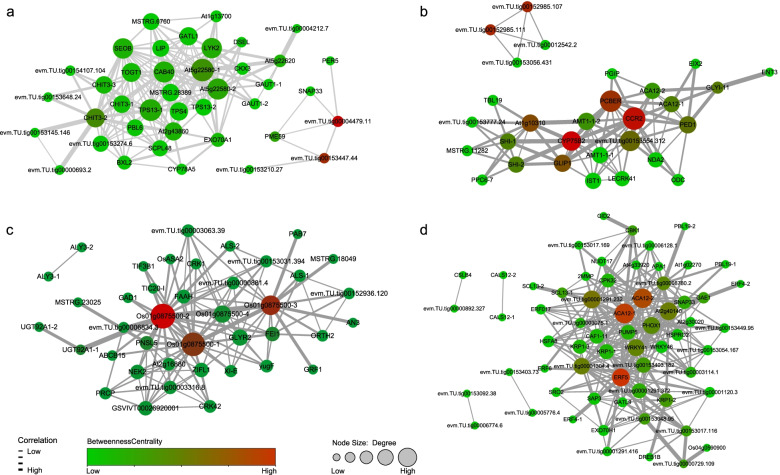
Visualization of the network connections among the most connected genes in the M13 (**a**), M12 (**b**), M7 (**c**), and M2 (**d**) modules. The size and red intensity of the nodes are correlated with degree and betweenness centrality, respectively

Finally, the co-expression networks of the M13, M12, M7, and M2 modules were filtered by a weight value greater than 0.15 and visualized (Fig. 6a-d). The genes with the highest betweenness centrality score in the M13 module were evm.TU.tig00004479.11 and evm.TU.tig00153447.44, which are non-specific lipid-transfer protein 2-like (LTP) and protein root initiation defective 3-like, respectively (Fig. 6a). The genes with the highest betweenness centrality score in the M12 module included CCR2, PCBER, and CYP75B2, which were cinnamoyl-CoA reductase 2-like, isoflavone reductase-like protein IRL, and cytochrome P450 71A1-like, respectively (Fig. 6b). The genes with the highest betweenness centrality scores in the M7 module were beta-galactosidase 10 (Os01g0875500–2, Os01g0875500–3, and Os01g0875500–1) (Fig. 6c). Finally, the genes with the highest betweenness centrality score in the M2 module were calcium-transporting ATPase 12 plasma membrane-type-like, ACA12–1 and ACA12–2, and the ethylene-responsive transcription factor, ERF105 (ERF5) (Fig. 6d). Further quantitative real-time PCR (qRT-PCR) analysis on the genes with high centrality score in the networks validated reliability of their expression patterns (Fig. S3), indicating the potential core regulatory roles of these genes during seed abortion.

## Discussion

The structural changes in the aborted seeds of *S. grosvenorii* at different phases observed using TEM revealed that the developmental abnormality began on 10DAF, gradually deteriorated until 20DAF, and then recovered (Fig. 1). These defects occurred when the levels of the endogenous plant hormones ABA and ZR were significantly down-regulated (Fig. 2). ABA and ZR were significantly decreased at 10–15DAF and 15–20DAF respectively. These findings indicate that 10–20 DAF is the critical period during seed abortion. ABA is considered one of the main hormones that regulate seed formation [32], and ZR can induce cell division and proliferation [28]. ABA application to fruit induces gene expression involved in cell wall modification, such as *PL, RGLyase*, and *βGAL1*, which are involved in pectin modification in bilberry fruit [33]. These effects were also described at proteome level for example, ABA treatment on Cabernet Sauvignon berries induced the protein amount of the xyloglucan endotransglycosylase, which function is related to cell-wall restructuring [34]. Decreased ABA content may lead to tissue abnormalities by affecting the structure of cell wall.

Transcriptome data at different developmental stages provide a large amount of data for the dynamic formation of triploid *S. grosvenorii* seeds. The results showed that the number of genes with significant changes in expression was highest during the early stages (5–10 DAF) of seed formation. However, relatively fewer genes changed significantly after 10DAF (Fig. 3a). Although fewer genes were differentially expressed at 15DAF vs. 10DAF and 20DAF vs. 15DAF, these genes were most likely associated with seed abortion since 10–20 DAF was considered as the critical period during seed abortion. Some genes involved in ABA metabolic process or responded to ABA were significantly downregulated at 15DAF vs. 10DAF. This was the case with the zeaxanthin epoxidases, ABA2 and ZEP, are involved in ABA metabolic process, and a predicted geranylgeranyl transferase type-1 subunit beta GGB is annotated as a gene respond to ABA (Table S3). The expression of the predicted UDP-glycosyltransferase 73C1-like UGT73C6 and predicted cytokinin dehydrogenase 5 CKX5, which are involved in zeatin biosynthesis, were decreased at 15DAF vs. 10DAF (Table S3). The predicted cytokinin dehydrogenase 6 CKX6 was decreased at 20DAF vs. 15DAF (Table S4). These results were consistent with the fluctuation of ABA and ZR.

During the embryonic development of a seed, a complete tissue structure must be established, and the cells of the embryo must divide and differentiate into cell types in an integrated manner. The basic body of a plant is formed during embryogenesis, and formation of most organs and tissues occurs in the late stage of the embryo [20, 35]. Studies on *Arabidopsis* seed mutants have shown that differentiation is regulated by cell division activity, which is inhibited by ABA [36]. It has been suggested that the cessation of G1 phase in cell division requires endogenous ABA [37]. A study of lupines found that a crucial switch occurred from the cis- to trans-forms of CKs, and the timing of this switch was important during development in determining whether a seed is viable or aborted [38]. Hence, seed abortion may be caused by abnormal embryo morphology due to ABA reduction, and ZR fluctuation at inappropriate time. Notably, genes involved in histone methylation changed significantly during 10–15DAF. In *Paeonia lutea*, the expression of some histone genes in common groups with normal active seeds was significantly higher than that in the groups with endangered abortion seeds. These histone genes are considered to be involved in seed abortion of *P. lutea* [39]. It was reported that histone monoubiquitination appears to regulate ABA levels in developing seeds through histone H2B monoubiquitination [40]. We suppose that changes of histone methylation may also affect ABA level and seed development.

The continuous downregulation of genes involved in starch and sucrose metabolism may lead to seed abortion. Sucrose is not only a nutrient, but also a signal that induces storage-related differentiation. It can induce premature mitotic termination and cell enlargement, activate the expression of storage-related genes in maize and *Vicia*, and promote storage activity in cotyledons at the transcriptional level and starch accumulation [41–46]. Under drought stress, the transcription and activity of invertase, which is important for controlling the ratio of sucrose to hexose, are reported to be reduced, thereby interfering with the sucrose use capacity and the ratio of sucrose to hexose, which disrupt maternal tissue development and eventually lead to seed abortion [47]. Sucrose and ABA also have similar effects on gene expression. Sucrose may increase ABA levels or enhance ABA sensitivity [48]. However, this hypothesis has not been proven. And it is considered that ABA may inhibit starch synthesis [49]. Here, the variation trend of plant hormone signal transduction is similar to that of starch and sucrose metabolism, which also reflects the potential mutual assistance between endogenous hormones and sugar metabolism.

Further analysis of the co-expression networks provided new insights into the regulatory mechanisms of ABA genes. The genes in the M13 module, which were negatively associated with the changes in ABA levels, were enriched in monoterpene metabolic processes and terpene metabolic processes (Fig. 5). Both monoterpenes and terpenes contain a series of terpenoids. In higher plants, terpenoids are substrates for several compounds, including ABA, cytokinins, and the phytol side chain of chlorophyll. In addition, ABA and the phytol side chain of chlorophyll, as well as many secondary metabolites, including monoterpene and terpene, are downstream products of isopentenyl diphosphate [50–52]. *CAB40* in the M13 module encodes the chlorophyll a-b binding protein of LHCII type 1. The expression level of *CAB40* was high and negatively associated with the fluctuation of ABA level at stage 15DAF, suggesting that terpenoids flux toward synthesis of the phytol side chain of chlorophyll. These results suggest that at 15DAF, the enhancement of the metabolic processes of monoterpene and terpene may lead to seed abortion by reducing substrate flow to ABA and cytokinins.

Previously, *LTP* was reported to be induced by ABA in the vegetative tissues of rice [53]. However, *LTP*, which had the highest betweenness centrality score in the M13 module, was negatively correlated with ABA based on our results, indicating that ABA may have different effects on the regulation of *LTP* expression in different tissues or developmental phases. In previous reports, two cytochrome P450 monooxygenases, CYP735A1 and CYP735A2, were shown to catalyze ZR biosynthesis in *A. thaliana* [54]. Here, in the M12 module, *CYP75B2* expression was negatively correlated with the level of ZR, suggesting a different role of cytochrome P450 monooxygenases in the biosynthesis of ZR.

It is also worth noting that genes in amino sugar and nucleotide sugar metabolism were in the M13 module, which was highly expressed at 15DAF and 20DAF. This is consistent with the STEM, wherein the expression of the genes in amino sugar and nucleotide sugar metabolism were peak at 15DAF and 20DAF. These results suggested that amino sugar and nucleotide sugar metabolism play important roles in seed development. In fact, it has been reported that 59 genes involved in amino sugar and nucleotide sugar metabolism specifically show different expression in two germplasms of sheepgrass (*Leymus chinensis* ((Trin.) Tzvel)) seeds, with different germination percentages at 14DAF [55].

## Conclusions

In conclusion, we found that ABA and ZR fluctuated significantly at 15 and 20 DAF respectively when the abortive seeds displayed structural abnormalities. Subsequent transcriptomic analyses provided us with a large amount of information, including the gene networks that may regulate the biosynthesis of hormones, ABA and ZR, as well as the metabolic pathways or biological processes that may be affected by plant hormones. Genes involved in starch and sucrose metabolism and ABA may influence each other in developing seeds. Changes in histone modifications may cause changes in ABA levels. The enhanced monoterpenes and terpene metabolism, and increased expression of *CAB40*, suggested ABA and ZR levels may be reduced due to reduction in the amount of their substrates. In addition, *CYP75B2* expression was negatively correlated with ZR, while *LTP* expression was negatively correlated with ABA. Changes in pathways and genes involved in the synthesis and metabolism of ABA and ZR may be associated with the fluctuations in their levels, leading to seed failure. These findings provide a strong theoretical basis and candidate genes for breeding seedless *S. grosvenorii* species using the genetic-engineering technology.

## Methods

### Plant materials and microscopic monitoring of seed abortion

The *S. grosvenorii* seedless 1 line used in this study was kindly provided by Mr. Xiangjun Jiang, Guilin Yiyuansheng Modern Biotechnology Co., Ltd., Guilin, China. We had got the permission to collect *S. grosvenorii* line. The line preserved in the germplasm resource nursery of the Academy of Agricultural Sciences of Guangxi Zhuang Autonomous Region, and identified by Prof. Xiaojun Ma. The triploid seeds of LHG were collected at consecutive intervals of 5 days (5DAF, 10DAF, 15DAF, 20DAF, 25DAF, and 30DAF). Transmission electron microscopy analyses were performed to gain insights into the submicroscopic structural changes in the tissues. The fresh tissues were fixed with Servicebio’s fixative solution, followed by fixation in 1% osmic acid and 0.1 M phosphate-buffered saline (pH 7.4) for 5 h. Specimens were then dehydrated with alcohol, embedded in SPI-Pon 812 epoxy resin monomer, thinly sectioned, stained with uranyl acetate and lead citrate, and examined under a transmission electron microscope (model HT7700; Hitachi, Japan). The study protocol must comply with relevant institutional, national, and international guidelines and legislation.

### Detection of endogenous plant hormone levels during seed abortion

The levels of endogenous plant hormones, such as ABA, ZR, IAA, and GA3, were determined using ELISA. Each sample was measured in parallel three times, except for the time point 15 DAF when samples were measured four times. Each replicate contained three seeds.

Briefly, the tissues stored in liquid nitrogen were thawed and homogenized at a temperature of 2–8 °C. Following the manufacturer’s instructions of ELISA kit provided by SHUANGYING Biological Ltd. (Shanghai, China), standards and samples were added to the wells of microtiter plates and incubated for 30 min at 37 °C. A total volume of 50 μL of horseradish peroxidase (HRP)-labeled detection antibody was then added and mixed gently, followed by incubation for 30 min at 37 °C. The 3,3,5,5′-tetramethybezidine dihydrochloride (TMB) was used as a substrate, and the reaction between the hormones (in standard and samples) and HRP was stopped with a reaction inhibitor within 15 min. The absorbance of each hormone was measured at a wavelength of 450 nm. After obtaining the hormone concentration in the sample, the hormone content in the sample was calculated.

### RNA extraction and sequencing

Three biological replicates, each containing three seeds, were collected at each stage. Total RNA was extracted at each stage using the TRIzol reagent kit (Invitrogen, Carlsbad, CA, USA) according to the manufacturer’s instructions. RNA integrity (RNA integrity score > 6.0) was measured using an Agilent Bioanalyzer (Agilent Technologies, Inc., Santa Clara, CA, USA). Eukaryotic mRNA with a polyA tail was enriched from total RNA using oligo (dT) coupled to magnetic beads and fragmented using fragmentation buffer (Ambion, #AM8740) to 200–400 bp. The first strand of cDNA was synthesized with M-MuLV reverse transcriptase, using fragmented mRNA as a template and random oligonucleotides as primers. The mRNA template chain was subsequently degraded by RNase H, and the second strand of cDNA was synthesized using dNTPs and DNA polymerase I. The double-stranded cDNA was purified with the QiaQuick PCR extraction kit (Qiagen, Venlo, Netherlands) and subjected to end repair, dA tailing, and adaptor ligation. cDNA fragments of approximately 200 bp were isolated using AMPure XP beads for PCR amplification, and the PCR product was purified using the AMPure XP system.

The quality and quantity of the library were analyzed using a NanoPhotometer® spectrophotometer (IMPLEN, CA, USA), Qubit® RNA Assay Kit with a Qubit® 2.0 Fluorometer (Life Technologies, CA, USA), and Agilent 2100 bioanalyzer and RNase free agarose gel electrophoresis. Based on the final libraries, a paired-end sequencing strategy (PE150) was carried out on the BGISEQ-500 platform (BGI Technology, Shenzhen, China).

### Global and differential gene expression analysis

After removing adapters, low-quality reads, and ambiguous reads from the raw data, the clean reads were aligned to the rRNA reference sequences (no mismatches allowed) using bowtie2 (version 2.2.8) [56]. The rRNA-depleted reads were mapped to the reference genome of *S. grosvenorii* using HISAT 2. 2.4 [57] with “-rna-strandness RF” and other parameters set as a default. Assembly of the aligned reads was performed using StringTie software according to the reference gff file [58, 59].

The number of reads mapped to each transcript was counted for each sample andnormalized to fragments per kilobase of transcript per million mapped reads (FPKM). The Pearson’s correlation coefficients of RNA expression between samples were calculated and principal component analysis was performed using R version 3.6.3. Differential expression analysis was performed using DEseq2 [60], and the genes with |log2FC| > 1 and FDR < 0.05 were determined as DEGs between pairs of adjacent stages.

To annotate the functions of the transcripts, BLAST (version 2.2.26) searches against the NR (ftp://ftp.ncbi.nlm.nih.gov/blast/db/) with an e-value cut-off of 1e-5 were performed. All the transcripts were then mapped to GO terms in the GO database (http://www.geneontology.org/) and KEGG database for the pathways [61, 62]. Gene numbers were calculated for every term/pathway, and significantly enriched GO terms/KEGG pathways in DEGs compared to the genome background were defined using a hypergeometric test. The calculated *p*-values were FDR corrected, and GO terms and KEGG pathways with FDR ≤ 0.05 were defined as significantly enriched GO terms and KEGG pathway respectively.

### Temporal analysis

Time-series analysis was performed using STEM [63] (p.191). Each mRNA was assigned to the model profile based on the expression pattern based on the correlation coefficient. The number of mRNAs assigned to each model profile and expected to be assigned to a profile were calculated by randomly permuting the original time point values, renormalizing the mRNA expression values, assigning mRNAs to their most closely matching model profiles, and repeating this process for a large number of permutations. The statistical significance of the number of mRNAs assigned to each profile compared with the expected number was then computed.

### Seed abortion associated gene co-expression network analysis

To explore the relationship between gene co-expression modules and plant hormone levels, WGCNA was conducted using the WGCNA package in R (3.2.2.) [64] (p. 559). The genes with FPKM > 0.3 were considered as expressed and used for WGCNA [65], and modules were obtained using the automatic network construction function, blockwiseModules, with default settings, except that the power was 18, minModuleSize was 50, and mergeCutHeight was 0.7. A module-trait relationship analysis was also performed using the module eigengene and the levels of ABA and ZR.

For genes in each module, GO and KEGG pathway enrichment analyses were conducted to analyze the biological functions of modules as described above. In addition, the network connections among the most connected genes (topological overlap above the threshold of 0.15) for the modules with statistical significance were further visualized using Cytoscape [66].

### qRT-PCR analysis

Ten core genes in the regulatory networks were selected for qRT-PCR to validate their expressions. First-strand cDNA was synthesized using the PrimeScript first Strand cDNA Synthesis Kit (Takara, Japan). Primer sequences were designed using the National Center for Biotechnology Information primer-BLAST (Table S13). qRT-PCR was performed by using SYBR Green PCR kit (Takara, Kyoto, Japan). qPCR reactions were performed using 1 μL of 1: 10 diluted cDNA and qPCR mix in a final volume of 10 μL. Thermal cycling conditions were 5 min at 95 °C followed by 40 cycles of 15 s at 95 °C and 60 s at 60 °C, and finally 5 s per step from 65 °C to 95 °C at a dissociation curve analysis. All reactions were performed in triplicates. The relative expression levels of target genes were calculated as fold change by normalization to 18S rRNA gene. The obtained results were analyzed using 2 − ΔCT method. The correlation between expression level revealed by RNA-seq and qRT-PCR was calculated accordingly to Pearson’s correlation coefficient.

## Supplementary Information

**Additional file 1: Table S1**. The expression level of all the annotated genes in S. grosvenorii genome.

**Additional file 2: Table S2**. The identifed DEGs between consecutive stages (10DAF vs 5DAF).

**Additional file 3: Table S3**. The identifed DEGs between consecutive stages (15DAF vs 10DAF).

**Additional file 4: Table S4**. The identifed DEGs between consecutive stages (20DAF vs 15DAF).

**Additional file 5: Table S5**. The identifed DEGs between consecutive stages (25DAF vs 20DAF).

**Additional file 6: Table S6**. The identifed DEGs between consecutive stages (30DAF vs 25DAF).

**Additional file 7: Table S7**. GO functional enrichment of DEGs (10DAF vs 5DAF).

**Additional file 8: Table S8**. GO functional enrichment of DEGs (15DAF vs 10DAF).

**Additional file 9: Table S9**. GO functional enrichment of DEGs (20DAF vs 15DAF).

**Additional file 10: Table S10**. GO functional enrichment of DEGs (25DAF vs 20DAF).

**Additional file 11: Table S11**. GO functional enrichment of DEGs (30DAF vs 25DAF).

**Additional file 12: Table S12**. The correlation coefficient between all the genes and modules in the coexpression network.

**Additional file 13: Table S13**. qRT-PCR primers used in this study.

**Additional file 14: Figure S1**. Principal component analysis (PCA) of the transcriptome across stages. **Figure S2**. Hierarchical cluster tree displaying the co-expression modules. **Figure S3**. qRT-PCR verified the expression of the core genes in the regulatory networks. R value in the top of each figure indicate Pearson correlation coefficient between relative expressions from qRT-PCR and transcriptome across stages.

## Data Availability

The datasets used and/or analysed during the current study are available in the NCBI Bioproject repository, [PRJNA773651].

## Abbreviations

ABA: Abscisic acid
ZR: Trans-zeatin
LHG: Luohanguo
GA: gibberellin
TEM: Transmission electron microscopy
DAF: Days after flowering
IAA: indole-3-acetic acid
GA3: gibberellin A3
ELISA: Enzyme-linked immunosorbent assay
FDR: False discovery rate
FPKM: Fragments per kilobase of transcripts per million fragments mapped
DEGs: Differentially expressed genes
GO: Gene Ontology
STEM: Short time-series expression miner
KEGG: Kyoto Encyclopedia of Genes and Genomes
LTP: Lipid-transfer protein 2-like
IRL: Isoflavone reductase-like protei
CYP: Cytochrome P450
ERF: Ethylene-responsive transcription factor
NFR5: Nod factor receptor 5
BSR2: Broad-spectrum Resistance 2
TPS4: Trehalose-6-phosphatase synthase S4
WGCNA: Weighted gene co-expression network analysis\
