# Supplementary material for: Genome-scale transcriptomic insights into the gene co-expression network of seed abortion in triploid Siraitia grosvenorii
Source: BMC Plant Biol. 2022 Apr 5;22:173. doi: 10.1186/s12870-022-03562-4 (PMC8981669; doi:10.1186/s12870-022-03562-4)
Supplement: Supplementary file 14 — Additional file 14: Figure S1. Principal component analysis (PCA) of the transcriptome across stages. Figure S2. Hierarchical cluster tree displaying the co-expression modules. Figure S3. qRT-PCR verified the expression of the core genes in the regulatory networks. R value in the top of each figure indicate Pearson correlation coefficient between relative expressions from qRT-PCR and transcriptome across stages. [file 12870_2022_3562_MOESM14_ESM.pptx]

## Slide 1
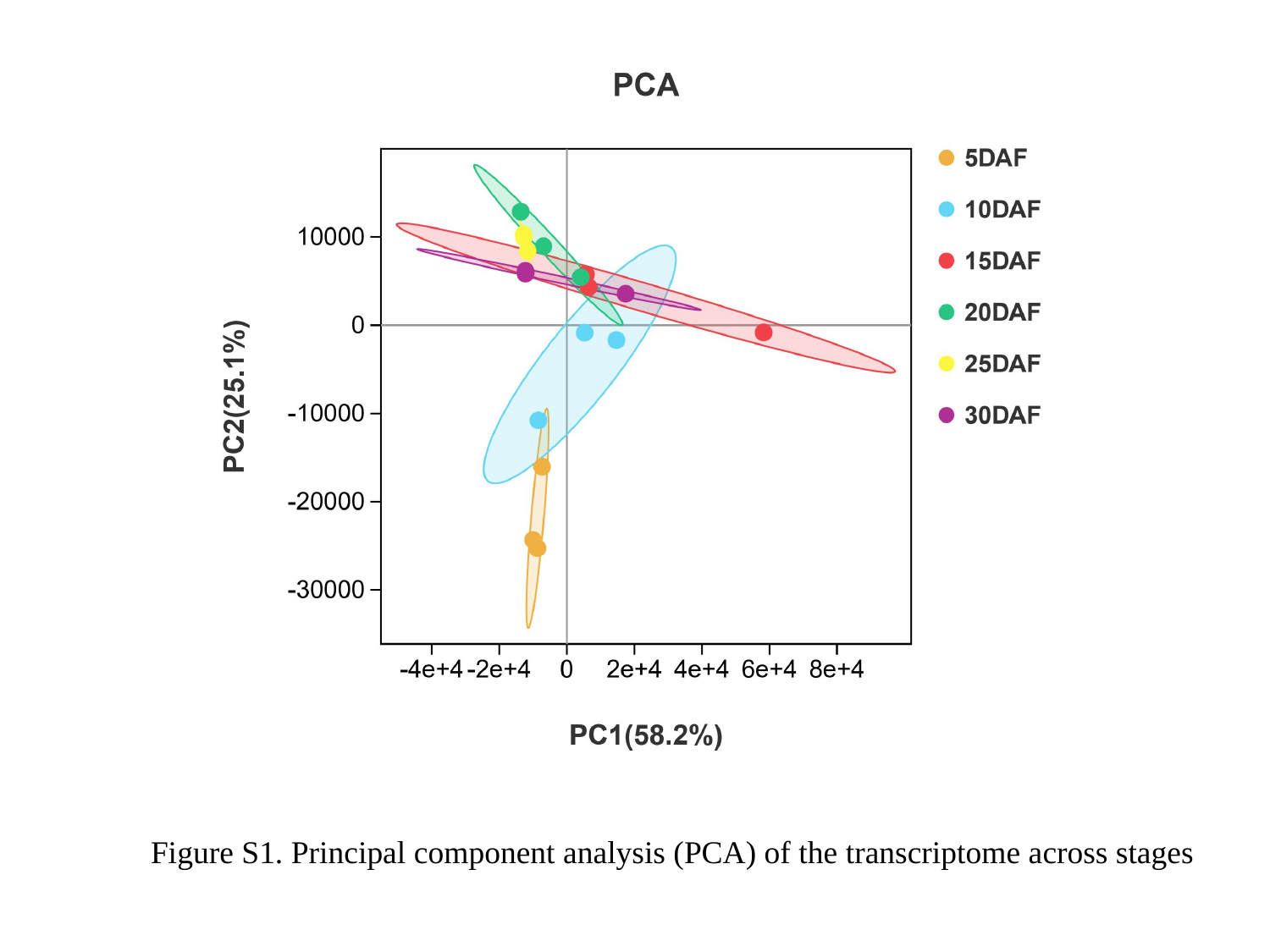

Figure S1. Principal component analysis (PCA) of the transcriptome across stages

## Slide 2
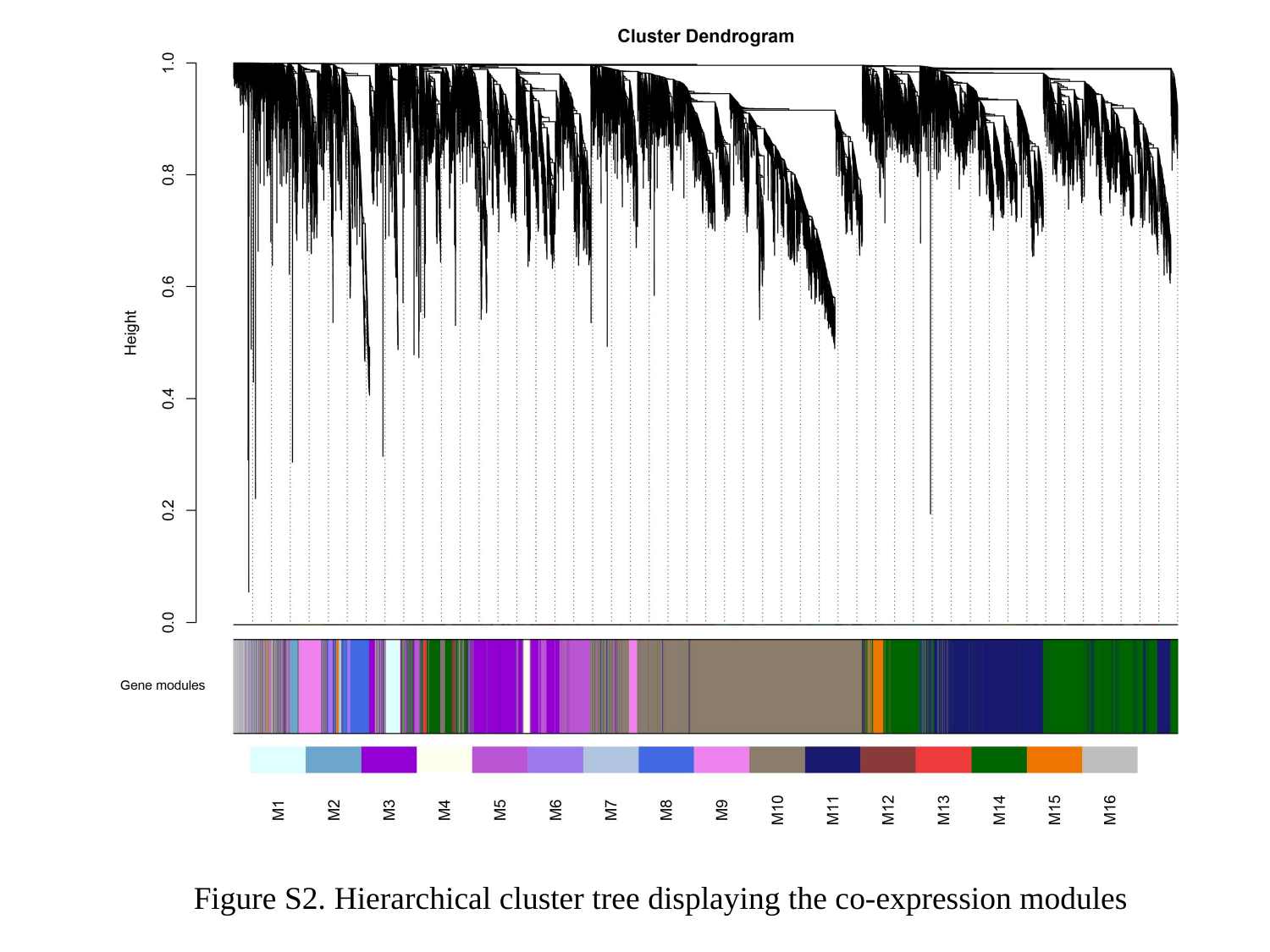

Figure S2. Hierarchical cluster tree displaying the co-expression modules

## Slide 3
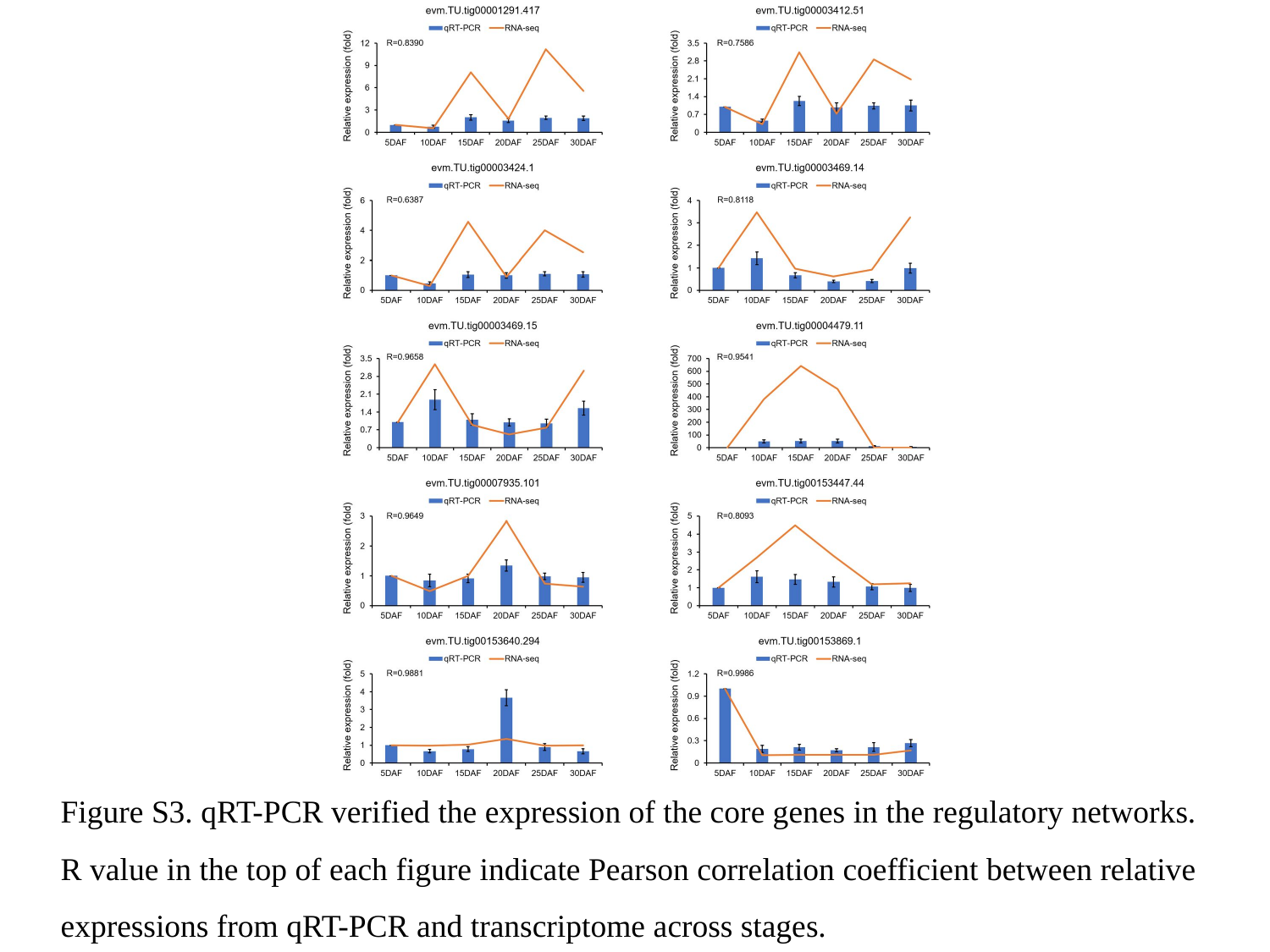

Figure S3. qRT-PCR verified the expression of the core genes in the regulatory networks. R value in the top of each figure indicate Pearson correlation coefficient between relative expressions from qRT-PCR and transcriptome across stages.
